# A Metagovernance Model of Innovation Networks in the Health and Social Services Using a Neo-Schumpeterian Framework

**DOI:** 10.3390/ijerph18116133

**Published:** 2021-06-06

**Authors:** Alberto Peralta, Luis Rubalcaba

**Affiliations:** Department of Business and Economics, Faculty of Business, Economics, and Tourism, University of Alcala (UAH), 28802 Alcalá de Henares, Spain; alberto.peralta@edu.uah.es

**Keywords:** metagovernance, public service, networks, PLS-SEM, collaborative innovation, sustainability

## Abstract

Health and social services (HSS) are now, more than ever, at the center of the debate of public policy. We are interested in studying the HSS services innovations from the networked-governance strategy standpoint. With this research, we contribute by analyzing the criteria leading to the formation of HSS public service innovation networks (HSS PSINs). These criteria are important because they may result in the much-needed empirical foundation of the metagovernance of public networks for sustainable innovation. Our analysis rests on neo-Schumpeterian interpretations of product, process, organizational, market, and input innovations, and their characteristics. By an empirical partial least squares structural equations model, we present here the relationships between those characteristics and HSS PSINs. Our intent is that these relationships become clearer, and help enhance HSS PSINs metagovernance—i.e., their control, democratic legitimacy, and accountability by public decision-makers. Hence, our research supports the voices for an extended use of networks for policy and service collaborative innovation for sustainability.

## 1. Introduction

The multiple cases of collaborative innovations to solve health and social services (HSS) problems throughout the COVID-19 pandemic have highlighted, yet again, the adequacy of scaled-up solutions to address complex, urgent, and wicked problems. In addition, networks and consortia have successfully addressed lasting and long-known problems of inequality, exclusion, social and natural ecosystems breakdowns, waste, and resource depletion. These multi-agent arrangements have effectively integrated patients, their families, and other multiple stakeholders to design and implement innovations [1].

However, these networks of public officials, clinicians, users, consultants, and the general public are also proving to be quite a challenge for elected politicians and senior managers. Their benefits, spanning from increased efficiency, effectiveness, and democratic legitimacy, are commonly overshadowed by their complexity—namely uncertainty, transaction costs, environmental and sustainable outcomes, and risk sharing [2].

These collaborative arrangements [3] are a “socially penetrative” mode of governance [2], producing largely unexplored effects, and affecting governance itself, outcomes, or innovation capability. These self-regulating, multi-agent entities aim to design and deliver new services and public policies—traditionally circumscribed to clinical specialists, researchers, healthcare suppliers, and decision-makers. The scarce academic literature on this field has named these associations public service innovation networks (PSINs) [3,4,5,6].

To our best knowledge, the academic literature has overseen the criteria featuring a PSIN and their effects on the governance, outcomes, and innovation capabilities of these networks. We believe that the understanding and visualization of PSINs components and their links with innovation and outcomes in the HSS sector are critical to improve their metagovernance, or “the role of the state in the oversight, coordination, and (…) resourcing of [these] governance arrangements” [2].

Accordingly, and from our empirical viewpoint, we want to contribute to the improvement of the metagovernance of PSINs by validating and presenting the significant components or criteria that build health and social services PSINs (HSS PSINs). We also investigate which types of innovations and outcomes HSS PSINs are connected with. Especially, we want to identify if HSS PSINs outcomes are more strongly associated with sustainability and sustainable goals than other public services PSINs (e.g., employment, mobility, or education). Finally, we want to contribute by empirically modelling HSS PSINs components and innovations. This model allows us to visualize their correlations and enhance the metagovernment of PSINs for democratic legitimacy and sustainable efficiency.

Consequently, we investigate and develop this model by using partial least squares structural equation modelling (PLS-SEM). This set of statistical algorithms has proved very sound in social research, linking complex theoretical models and original data. Throughout our research, this complexity, which PLS-SEM resolved successfully, entails a multilevel investigation of HSS PSINs composite components or criteria—to bring on a hierarchical-component system or model [7,8]. Additionally, we need to validate the correlations between the PSINs-forming criteria and the PSINs’ outcomes and innovations; previously, we ensured the equal interpretation of both criteria and outcomes by our sample research participants, i.e., the absence of measurement invariance [9].

With this research, we conceptualize the specific components of HSS PSINs, contributing to the theory of network metagovernance in the public sector. From a practical standpoint, we confirm the implications of PSINs (and their components) for innovation development and their sustainable governance. In particular, we provide concrete evidence to encourage the legitimate and accountable governance of networks by public decision-makers.

Next, we review the theoretical underpinnings of the components of HSS PSINs and their effects on public service innovation and sustainability. Then, we present our methodology and offer our results and findings. Finally, we finish our paper with a discussion and conclusions section.

## 2. Theoretical Framework

### 2.1. Patients, Public, and Other Stakeholders’ Involvement for Innovation

Under the pressure of the consecutive recent economic crises and the current COVID-19 pandemic, many governments are looking at multi-actor arrangements, or networks, with renovated interests. They seem a source of unconventional innovation with the potential benefits of increased trust, efficiency, and democratic legitimacy. The fact is that these networks constitute a form of governance that has been profusely used and documented in the past [2,10], but with little reflection in current research and references [3,11,12].

These networks seem to be particularly relevant in the health and social services (HSS) sector [1,13]. This sector combines the characteristics of two important domains of public intervention to define a broader-scope type of services. The HSS intend to improve the health of communities and populations [1] and to reduce waste and inequities. As a consequence, their recent developments spawn from the multiplicity of agents participating in their scaling-up, i.e., the multiplication of the impact of innovations to benefit more people, reduce more waste, and foster more sustainable policies in the longer run. Specifically, we understand HSS scaling-up as, for example, merging clinical products and services, and integrating patients and their families in service design and delivery, or shifting the mindset of elected politicians and senior public managers to support lay public and non-specialists co-developing HSS innovations with specialists and suppliers. This scaling-up will eventually reduce the harm in HSS ecosystems.

Nevertheless, those at managerial and political positions often opt for top-down developed solutions. Researchers, public officials, and conventional clinical teams feel more secure following New Public Management (NPM), or even neo-Weberian state (NWS), approaches to innovation of public services. After NPM, politicians, managers, researchers, and teams become efficient by entering in market competition and using private sector managerial modes of governance [14]. Following NWS, the same teams become more efficient through an increased responsiveness to patients, the general public, and other users of health systems [10]. In either case, the values, interests, knowledge, or trust of other, non-team stakeholders are easily ignored or realized much later.

Many have criticized both approaches (e.g., [5,10,15,16]) as they nurture “in-house” innovations. These are usually proposed programmatically by politicians, controlled conventionally by public managers, and executed bureaucratically by trusted teams of civil servants and public researchers. Others add to these critiques the excessive technical, medical orientation of these innovations [17], which in many cases transform the essence of public health services into a sort of product, similar to a drug or pill that can be used independent of context and social or environmental consequences.

Alternatively, the collaborative approach [10,11,18], which places the patients and citizens, and now the sustainable development, at the core of the healthcare systems, might be a more efficient approach to the innovation of HSS services. Under this collaborative approach, the nontechnical features of innovation become even more relevant than their technical equivalents. The problems to be solved are informed and understood from different, non-conventional sides—including the social and environmental sides—through the commitment of multiple, non-conventional, even lay actors. Additionally, this approach advances the innovations themselves, as the multi-agent practice matures along its life cycle. Finally, these collaborative innovations garner stronger support and wider ownership and accountability [10], and result in greater citizen empowerment, societal equity, and government trust and legitimacy.

The collaborative approach, associated with the New Public Governance (NPG) [19], or Public Service Logic [19], explicitly acknowledges the differentiation between technical and nontechnical characteristics of HSS innovation. This demarcation is realized throughout the preparatory, execution, and translational phases of the scaling-up of innovations [1]. In every phase, the involvement of the patients and rest of stakeholders should be meaningful and equitable, carrying an equal weight as that of the medical and research partners. Precisely, it is the empowered involvement of patients and citizens that gives the collaborative approach its broader scope and greater conscience of the ecological and social (i.e., nontechnical) aspects of innovation.

Nonetheless, the greatest nontechnical challenge of these multiagent arrangements is their governance [10,11] and their implications in terms of the regulation, control, coordination, and resourcing by the state, i.e., their metagovernance [2]. More importantly, the bottom-up innovations created by networks must fulfill the legal requirements of legitimacy and accountability demanded by governments and elected politicians as carriers of both mandates [20].

HSS networks, on that account, must give proper solutions that address public health and social issues, or any other public health concern, bringing government in and avoiding the state’s hollowing out [2,21], i.e., the offloading of functions to the networks, or “shadowed hierarchies,” by a weakened state [22]. If the networks’ innovations are to improve services and public value through the diffusion of HSS innovations [10], then they must first leverage their bottom-up approach bringing the decision-makers in, i.e., preventing the state’s hollowing out.

Consequently, we found repeated calls (e.g., [16,23,24,25]) to investigate those nontechnical characteristics of innovation networks operating the HSS sector. These calls request a closer look at the integration of technicalities with their human choices, social and civic characteristics and competences, environmental conditions, and governance and metagovernance implications. This integration drives our analysis of public sector innovation networks (PSINs) [3] as a collaborative strategy in the HSS sector.

Hence, with this research, we intend to identify the relevant technical and nontechnical criteria of PSINs operating in the HSS sector. These criteria will set specific indicators for PSINs’ governance. Along with this goal, we aim to describe how these criteria produce the types of outcomes and innovations that the state and elected politicians seek, in order to fulfill their legitimacy and accountability mandates and political programs. These correlations will provide evidence for an improved metagovernance of HSS PSINs.

We position our research, then, as a study of the “networked governance” strategy [4,26] of PSINs in the HSS sector (i.e., HSS PSINs). This strategy has reportedly shown its positive effects on innovation [27,28,29], addressing social and ecological goals [30,31,32,33] and preventing the hollowing out effect [34]. It also strengthens all stages of innovation and scaling-up [35]. We propose to study these HSS PSINs governance and metagovernance, in a neo-Schumpeterian framework [16,36,37] defined by organizational, product, market, process, and input dimensions [38].

Gallouj and his colleagues defined PSINs [3] as “collaborative arrangements” of multiple actors, aiming to co-create public value through new services and system hubs. The collaboration between these actors is far from being understood as unanimous consent, for it is unrealistic in a context where actors with different assets and commitments meet. Rather, collaboration might refer to the engagement of different individual actors, representing themselves or someone else, to develop joint solutions based on agreements achieved through the self-management of disagreements and dissent [10]. Functionally, PSINs are a mode of coordination of these actors over the stages of innovation development, but also, they are a means of connecting the partners with the HSS institutions and their governments [39,40].

Following Desmarchelier and colleagues [4,18], and the reports from the COVAL project and others [41,42,43,44,45,46,47], we drew on the criteria that distinguish HSS PSINs and their relationship with innovation outcomes and types, and grouped them in categories. These second-order constructs or categories describe the agents in HSS PSINs and relevant descriptors of their interactions. We were also interested in the criteria describing the types of projects, engagement, or life cycle of these PSINs. The type of arrangements and how the partners come together were also of interest. Finally, the nature of the targeted innovations and outcomes were relevant, as they show the potential ties between PSINs and their metagovernance.

### 2.2. The “Neo-Schumpeterian” Framework of HSS PSINs

The multi-agent framework from Windrum and Goñi [16], based on Schumpeter’s approach to innovation [38], is a valued source to recognize and organize the criteria that characterizes PSINs. Initially, their “product innovation” interpretation alluded to the introduction of a new service, or the incremental improvement of an existing service. From a metagovernance stance, they explicitly described how decision-makers or users [16] (p. 657) might initiate either type of product innovation. To these, we add that such types of innovation can stem from the PSINs themselves and, specifically, from the collaborative efforts within the PSINs. These, as independent entities from their partners, have distinct combinations of voices and assets, and above-average capacities to act on every stage of innovation development.

The “process innovation” indicated the changes in the production or delivery of a service. This type of innovation originally entailed, the same as product innovation, a base of technical (medical) features and stakeholders: clinicians, doctors, specialists, nurses, researchers, and provisioners. However, under the collaborative interpretation of the process innovation, other features, namely social and environmental, are equally relevant: leadership, implicating lead users [48], decision makers, and eco-stakeholders; culture and mindsets, seeking social efficacy [13]; or usage of innovation tools and practices, relating to the governance mode or paradigm of the government [49]. From a metagovernance perspective, process innovations might be better controlled through a set of new and conventional metrics. These metrics comprise technical process indicators and nontechnical indicators, which exhibit the new processes’ technical, social, and environmental consequences.

The introduction or change of new organizations or organizational competences, or the recast of established organizations, or parts of them, were addressed under the “organizational innovation” dimension. PSINs are themselves a form of organizational innovation. However, it is one that fits with difficulty in the conventional organizational definition of innovation measured through organizational performance. PSINs are seldom constituted to improve technical efficiency. Rather, their governance is much more interested in managing disagreements and dissents, lowering barriers to communication and interaction, and empowering partners to enrich each innovation stage. These interests come before organizing the partners in a “most efficient way”. At the very least, the metagovernance of HSS PSINs must recognize the implications of their different rationality, legitimacy, and accountability [13], and reconcile these with the legal (state’s) legitimacy and accountability.

HSS PSINs and their collaborative strategy are certainly prone to innovating existing markets, per Schumpeter’s “market innovation” dimension. Clearly, and in the context of the collaborative strategy represented by PSINs, this original dimension might be understood as the “ecosystem innovation” dimension. This dimension stems from the “system innovation” concept [50,51] without leaving out the specificities of the broader and deeper scope of the collaborative strategy. We can emphasize some of them, paralleling other collaborative innovation research [1]. On one hand, the actors in these new ecosystems are the actual partners of PSINs—at least, some representatives of them are. They are any individual or group who is responsible for or affected by any combination of health- and social-related issues causing in them an unfulfilled need or unresolved pain. On another hand, HSS PSINs nurture product and process innovations, bringing on new or updated outcomes and innovation types, and new or updated organizations. The combination of both actors and HSS PSINs innovations should be enough to innovate existing (maybe depleted) ecosystems.

In addition, their context, where actors and outcomes of HSS PSINs meet, is in constant evolution due to the nature of the HSS sector. At its core is the acceptance of the impossibility of reaching a risk-free wellbeing [52], particularly in endangered communities or ecosystems. This realization also affects HSS PSINs metagovernance, as the ecosystem represents the largest scope where PSINs dwell and serve. It is where the already stated metagovernance precept of bringing in the government and the rest of the stakeholders, including the environmental stakeholders, realizes its full potential. Hence, the new ecosystems are the primary playfields of HSS PINS, where they must diffuse their innovations and produce their most visible impacts.

Finally, HSS PSINs actors bring in a broad and deep set of (new) assets, which renovate existing inputs and even carry new inputs, as a form of “input innovation” [38]. This dimension was traditionally assimilated to technological inputs. Besides them, with HSS PSINs, it entails new nontechnological inputs: from opinions to knowledge; from participation to commitment. Actors in HSS PSINs balance out their roles as provisioners with others such as lead users, minority affected, or empowered decision-makers. From a metagovernance stance, the new inputs of PSINs result from that duality of the PSINs actors. This is a differentiating element where the new inputs come in from the PSINs broader base of partners, including politicians and senior management, balancing all of them out with the traditional technical inputs.

**Figure 1:**
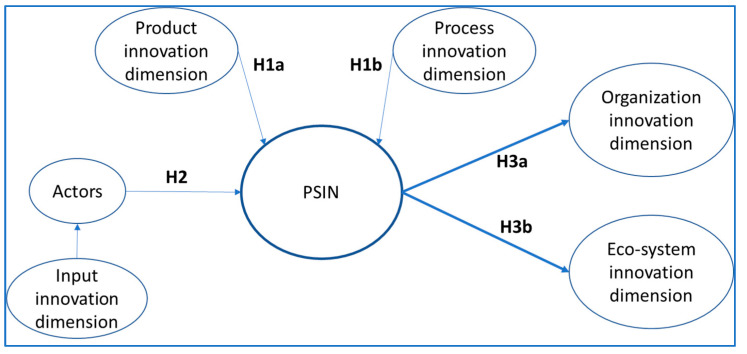
Research model.

Figure 1 illustrates our research model based on the interpretation of the multi-agent neo-Schumpeterian framework we just described.

### 2.3. Research Hypotheses

From the “product innovation” and “process innovation” [38], we theorize that both types of innovations inspire the actions, projects, measurements, and engagement in PSINs [16]. Thus, they must be part of the metagovernance analysis facilitated by our model. Consequently, we hypothesize:

**Hypothesis** **1a** **(H1a)**. *Product innovations, resulting from different types of projects, will positively influence HSS PSINs, and the nontechnical projects, or technical–nontechnical projects, are more strongly related to HSS PSINs.*

**Hypothesis** **1b** **(H1b)**. *Process innovations, measured by unconventional indicators and higher engagement levels, will positively influence HSS PSINs.*

The “input innovation” dimension [38] motivates another perception of the multi-actor arrangement of PSINs, beyond mere testing fields, as in focus groups. These actors share a dual role because they are both providers and users [16]. Under these conditions, the development of innovations is now informed through a constantly evolving combination of technical requirements and governance of several voices, equally represented and weighted. Within these voices, although not prominent, we count the politicians’ and decision makers’ opinions, assets, and legitimate mandates [2]. We, therefore, theorize:

**Hypothesis** **2** **(H2)**. *The extended base of actors of HSS PSINs, including clinical specialists, researchers, patients, the general public, and any other interested stakeholder, will positively influence HSS PSINs, and the influence will be equally weighted across the stakeholders.*

The “organizational innovation” and the “ecosystem innovation” dimensions outline the outcomes of PSINs [40,51,52]. Whether they are measured with output indicators, or they are presented as innovation types, HSS PSINs success and their innovations’ diffusion might be understood and presented as a step forward, either organizationally or as a new ecosystem [16]. Needless to say, these innovations should be in accordance with the democratic legitimacy and accountability, and political aspirations of public decision makers [13]. Consequently, we hypothesize:

**Hypothesis** **3a** **(H3a)**. *HSS PSINs will positively influence organizational innovations, and their outcomes will be measured with conventional indicators as well as new nontechnical indicators.*

**Hypothesis** **3b** **(H3b)**. *HSS PSINs will positively influence ecosystem-type innovations, and the effect will be stronger with nontechnical innovations.*

To analyze the multiplicity of interactions and the effects described in Figure 1, we decided to use partial least squares structural equation modelling (PLS-SEM). PLS-SEM is suitable to address the challenge of assessing the more than one hundred variables of our model, their interactions, and effects. We describe the methodology in the next section.

## 3. Methodology

Our research is exploratory [8,53,54] and started from a review of the scarce literature on the intersection of services innovation, cross-sectoral networks, and sustainability. We then decided to make a comparative analysis of HSS PSINs (i.e., PSINs operating in the health, aging, women, excluded populations, minorities, and child- and youth-care) and other public sector PSINs (Other PSINs) (i.e., education and training, transportation and mobility, environment and urban issues, and employment). This analysis only included PSINs oriented to sustainable (economic, social, and/or environmental) goals, perceived by their actors (i.e., our sample participants).

We accomplished our research goals by initially designing a questionnaire that was tested and piloted as described in Section 3.3. The completed questionnaires (n = 214) were collected by the research team themselves. We analyzed the survey’s results using SmartPLS (V. 3.3.3) (SAGE Publications, Inc.: Thousand Oaks, CA, USA) [55]. In the following, we provide further details on the survey instrument, data collection, demographic descriptive variables, and model analyses.

### 3.1. Survey Design and Measurement of the Constructs

**Figure 2:**
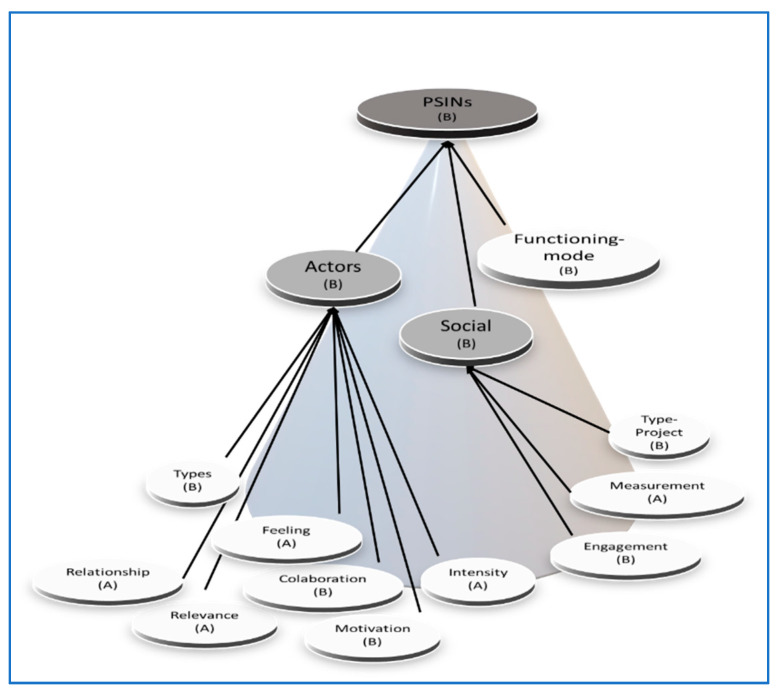
The research hierarchical model. Lower (first)-order components (white background) and higher-order components (second-order components are in light-grey background and the third-order component is in dark-grey background) of the PSINs model—each component is represented with its measurement mode (A or B).

To the best of our knowledge, this is the first analysis of our theoretical constructs. We created the questionnaire’s items out of the work of Gallouj and colleagues [3,17,18] and the COVAL project research reports. Thus, we knew little about their measurement specifications, i.e., whether we should measure criteria from their observable effects (mode A), or the other way around, as composites of observable determinants (mode B). Hence, we tested and validated their measurement modes [56,57], following the Confirmatory Tetrad Analysis suggested by Hair and colleagues [7] and Jarvis and colleagues [56]. It resulted in the identification of the measurement modes of the criteria (Figure 2). Finally, we confirmed content validity of the criteria by supporting their modes with the underpinning theory.

Our description of PSINs included two types of variables: criteria, or latent composite variables, which represented the theoretical dimensions of PSINs, and items, or observable manifest indicators, which were the questions in the questionnaire. Criteria were linear combinations of the items that we chose to study, based on our theory and case studies.

### 3.2. Hierarchical Component Model

In our PSINs model, these criteria made up the first, second, and third layers of composites, hierarchically ordered in a hierarchical component model (HCM) [58]. Thus, we reduced the number of relationships, fitting the research model in Figure 1. At length, the HCM facilitates the visualization of the constructs relationships, and is more precise (parsimonious).

Figure 2 illustrates our HCM. The research model’s lower-order components (LOCs) captured the theoretical dimensions of the higher-order components (HOCs). The layer of first-order LOCs (white background composites in Figure 2) formed Social and Actors (as theoretically indicated by [3,17,18]). Social and Actors (light grey background) added to Functioning-mode to shape PSINs ([3,18]). PSINs is the third-order HOC (dark grey background).

**Figure 3:**
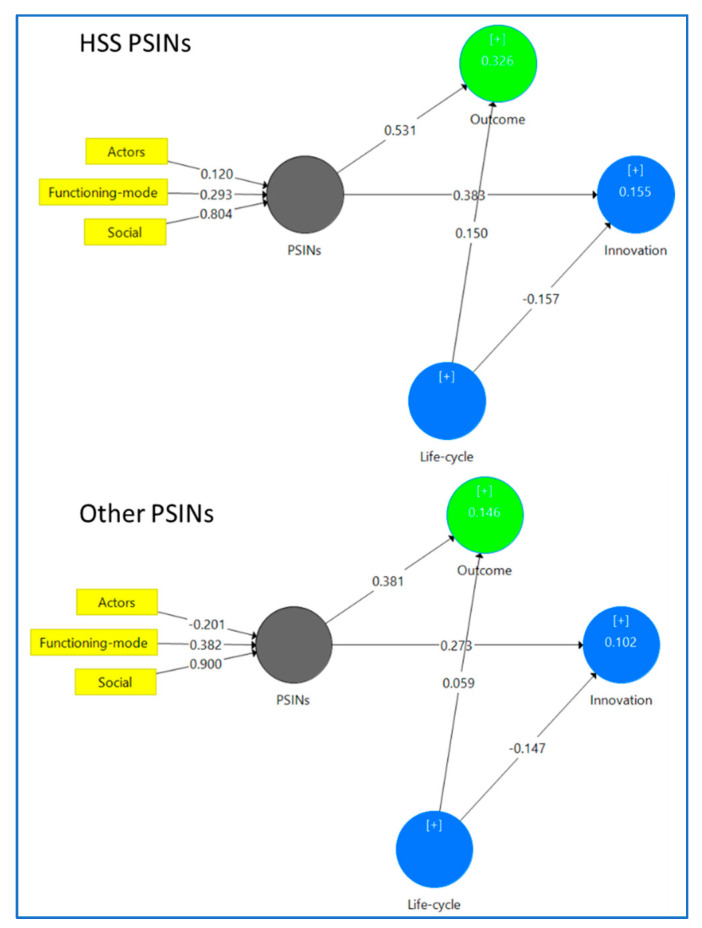
Empirical research model. Upper figure: HSS PSINs. Lower figure: Other PSINs.

The conceptual model in Figure 1 translates then, after the PLS-HCM aggregation routine [58], into the empirical model in Figure 3.

### 3.3. Survey Administration and Demographic Distribution

Our initial pilots to test the survey tested its unsupervised adaptation to the respondents (e.g., anonymity, duplicity prevention, improved accessibility, or 24 × 7 uptime). In addition, we tested the questionnaire cognitively [59] with 12 random members partnering with actual PSINs from the health and social services.

We extracted the contact information of our respondents from social network sites. In these publicly accessible sites, public servants, managers, and employees of HSS NGOs openly declare their jobs and we picked only those working in any of our target sectors. From early April to late June 2020, we contacted 2791 individuals fulfilling our research requisites. Then, 1034 of them reacted to our invitation to participate in the research, and a randomly selected population of 565 received our on-line questionnaire built on Limesurvey (V. 2.73.1). Finally, we retained 214 completed questionnaires for our analyses.

Using G*Power [60], we estimated the minimum sample size in 55 respondents (F-square: 0.015; alpha: 0.5; power: 0.8). The minimum sample size was 89 with more stringent parameters (F-square: 0.015; alpha: 0.5; Power: 0.95). Our sample of 214 cases, located across Spain, met this requirement. We selected Spain for our study, partially because this research is grounded on the work that we led for the COVAL project. Additionally, Spain is a multi-tiered conglomerate of public administrations. We decided to limit them to four tiers: municipal, regional, cross-regional, and national (federal). The multi-layers make the country better suited to study networks and connections within public services (see [61,62,63]). Finally, our data were gathered throughout the first wave of the COVID-19 pandemic, so we limited our geographical scope to prevent unnecessary physical exposure of the team members.

Table 1 shows the final distribution, per geographical scope (see other distribution in the Appendix A), of our survey participants.

Table 1 shows that the HSS share weights a little more in our demographic distribution (56%). From our respondents’ perspectives, few PSINs and entities care about environmental matters/goals, coupled with social and economic (11%). Most of our respondents are females (72%), aged evenly between 26–45 and 46–65 (50% each range). We have also a fair distribution of participants per education level (higher/other education: 51%/49%).

### 3.4. Model Analyses

For our model analyses, we decided to use partial least squares structural equation modelling (PLS-SEM) routines and algorithms for two main reasons: we needed a sound aggregation mechanism for our 114 questionnaire items that could validate the validation of the compositional invariance of the sample out of the aggregated model. First, it should help assemble the aggregated model, so that we could work out our sector comparisons with our limited sample. Subsequently, the tool must help us validate that the sample respondents understood the aggregated components in the same way—this is a required step to then perform our segment comparisons.

Additionally, PLS-SEM demonstrated its “ability to [create] independent latent variables directly on the basis of cross-products involving the response variable(s)” [8]. Furthermore, Henseler and his colleagues [64] recommended PLS path modeling “in an early stage of theoretical development in order to test and validate exploratory models,” which precisely is the stage of our research string.

The relationships of the first-order higher-order components (HOCs) with their items are either reflective (mode A) or formative (mode B). Hence our hierarchical component model (HCM) is of the Reflective–Formative type for the second-order HOCs, and Formative–Formative type for the third-order HOC. The relationships of the HOCs with their lower-order components (LOCs) are formative in all cases. In this scenario, Hair and colleagues [28] suggested a two-stage HCM analysis, repeated with every component.

In the first stage, we determined the linear relationships (paths) of the indicators and their criteria (LOCs), and calculated the latent (standardized) scores of the sample. The calculation of the paths created the scales for every LOC, aggregating indicators according to the extant theory. We adopted the strategy of retaining as many theoretical indicators as possible and essentially ensuring the criteria’s internal consistency.

To assess the criteria’s consistency, we used the weighting scheme in the PLS algorithm and bootstrapping [58] included in Smart-PLS 3 (V.3.3.3) software [55]. The (measurement) assessments included tests of reliability, validity (convergent and discriminant), collinearity, and relative contribution (effect size and significance) of each criterion, according to its mode of measurement. The results are in Table 2. We ended the stage after the calculation of the latent variable scores (LVS), and proceeded to the second stage.

In the second stage (Figure 2), we calculated the paths between the first-order LOCs and their HOCs (Social and Actors) in the same manner as the first stage’s paths. Extending the recommended analysis due to the third-order HOC, we needed a third stage (Figure 3). Thus, we repeated the calculation of the paths from Social, Actors, and Functioning mode to PSINs, using a new set of LVS from the second stage.

We then moved ahead to assess the predictive capabilities of the model. To effectively validate them, we assessed the HCM’s goodness of fit with other tests [65] (Table 2): the significance of the HOCs (and LOCs) path coefficients, the R^2^ values, the predictive relevance Q^2^, and the q^2^ effect size.

Finally, we strived to validate the differences between HSS PSINs and Other PSINs. To perform any contrasts on the two segments, Henseler and colleagues [9] advised to ensure measurement invariance before proceeding to the multigroup analysis (segments comparative). (If measurement invariance is confirmed, the potential group differences can be explained from variations in structural relationships, leaving aside differences from content or the groups’ meaning of the constructs. Demonstrated measurement invariance supports the conclusions and validity of multigroup comparisons.)

We validated measurement invariance in two stages. Initially, our MICOM procedure [9] (refer to the Appendix A) validated measurement invariance between the health sector participants and the social services participants. Therefore, we could safely assess—with the PLS-MGA technique [52]—that these segments carried insignificant differences between them. This validation permitted us to pool them into the HSS PSINs segment. This first stage was repeated with the Other PSINs segments.

The second stage in the multigroup analysis was the evaluation of the differences between HSS PSINs and the Other PSINs. Again, we should initially validate the measurement invariance between the two segments. We continued to the PLS-MGA to assess the differences between the two segments (see Table 2).

### 3.5. Control for Common Method Variance

Our survey respondents self-reported on their behaviors. Hence, common method variance (CMV) might potentially affect the validity of our conclusions [66]. CMV can bias the measures by the method of measurement rather than by the theoretical constructs represented by the measures [66,67]. Practically, CMV represents “the amount of spurious correlation among the variables that may be generated by utilizing the same method (i.e., survey) in order to measure each [dependent or independent] variable” [66]. To confirm the absence of the CMV effect, we followed the Measured Latent Marker Variable [68] (p. 146) recommendations for pre- and post- controls. Controls included (ex-ante) in-survey measurement of unrelated items drawn from the X1 version of the Malowe–Crowne Social Desirability Scale of Fisher and Fick [68,69]; different formats of response, e.g., random presentation of the items, negative wording of some items; anonymity of participants. We also used ex-post controls using the Construct Level Correction Approach [68,70].

## 4. Results

After confirming the absence of CMV in our sample, we created the scales for every first-order criterion and assessed their reliability and validity. We ensured the first-order criteria’s internal consistency as shown in Table 2. After securing the reliability and validity of our first-order criteria, we estimated the paths between the second- and third-order constructs (see Figure 3). From this second stage, we dropped Feeling, Intensity, and Relationship as criteria for Actors due to their nonsignificant paths.

The variable MODORG02[MO09] allowed us to split our sample into the two segments (sectors) under study. We successfully established full measurement invariance for our health and social services segments, thus we could group them into a new segment, the HSS PSINs segment. In the next stage, we could also validate the measurement invariance between HSS PSINs and Other PSINs, which was the requirement to assess their path differences with the PLS-MGA. The PLS-MGA resulted in nonsignificant differences between our target sectors (refer to Table 2 and the Appendix A).

Our model’s predictive capabilities are moderate (refer to the R^2^ scores, Table 2) with medium effect size (refer to Q^2^ scores and q^2^ sizes, Table 2), at best. The Outcome coefficient of determination of the HSS PSINs segment (R^2^: 0.326) is much larger than the Other PSINs (R^2^: 0.146). Meanwhile, the Innovation coefficients of determination are much weaker in both cases (HSS: 0.142; Rest: 0.102).

## 5. Findings

The assessment of the second-order components shows that Social, Actors, and Functioning-mode are relevant criteria, or dimensions of the PSINs concept. Social is the strongest influence for the HSS PSINs as well as the Other PSINs.

The Social criterion of HSS PSINs has three dimensions: Engagement, Type-project, and Measurement; path differences with the Other PSINs are unnoticeable. The three criteria aggregate the underpinning theory regarding social aspects driving the relationships of the networks’ partners. It also allows us to investigate H1a and H1b. The items forming Engagement, which is the strongest effect on Social, describe how the citizens as network partners help improve the assessment of the user needs and their satisfaction pre- and post- innovation; and the engagement and evaluation of users improves with market research techniques. H1b is then partially confirmed.

The Type-project dimension groups items describing the projects of HSS PSINs: integration of products and services, design of public services, and new ways of achieving HSS goals different from established and bureaucracy. H1a is then confirmed. The Measurement dimension produces a very small positive effect, in comparison with the other two. It reflects the traditional need of public organizations to monitor costs, returns, or value-added, and to concentrate on conventional productivity or efficiency measurements.

The Actors criterion is far weaker than we expected. In case of Rest PSINs, it is not even a dimension, due to its low significance. There are two components of Actors (having dropped Motivation and Relevance due to their irrelevance). Collaboration reflects again users, citizens, and other partners (consultants, other civil servants) in idea generation and prototyping and even the involvement of users and citizens in co-production/co-implementation. As expected, the Types component reflects the intense participation of citizens in HSS PSINs, and to a lesser extent universities and research centers. Strikingly, none of the other potential partners were significant. H2 is then not confirmed.

Their Functioning Mode also shapes HSS PSINs and the other sectors’ PSINs. Its effect is similar in both types. PSINs are theoretically forged over trust, and this is the main component of HSS PSINs. Based on trust, partners in HSS PSINs formalize it in contracts. In the Rest PSINs, trust is less evident and significant, residing their relationships merely on the network contracts.

With the model’s third-order component, PSINs, we can study the influence of networks and their life cycle on their outcomes and innovation. Although the model explains a small portion of both for our two target sectors, it is stronger for HSS. Significant outcomes of HSS PSINs, positively affected by PSINs and their life cycle, are a larger number of citizens able to access a new service, higher implementation time, higher design time, higher service quality, and higher employee satisfaction. The outcomes are similar for Other PSINs, except that Other PSINs seem to negatively affect the user experience with their new services. H3a is then confirmed.

HSS PSINs’ positive correlation with their innovation types is lower than outcomes (Other PSINs are a similar case). However, their life cycle is inversely related to the production of innovation, meaning that the younger the PSIN, the more innovative its services. HSS PSINs are connected to changes in how people think, new concepts or ideas, organizational changes, and new strategies or policies. The innovation types are similar for Other PSINs. H3b is then confirmed.

Although we have emphasized some of the differences between our target sectors, it is notable that our sample rejected the hypothesis of different formations of PSINs. That is, we found no differences in the criteria or higher-order components paths between our HSS and Other types of PSINs. This might imply that, besides being an organizational innovation breaking conventional boundaries and silos, PSINs are quite homogeneous across sectors, at least within the dimensions we have controlled in this research.

## 6. Discussion and Concluding Remarks

With this research, we wanted to describe the characteristics or criteria of public service innovation networks (PSINs) [3] for sustainability in the health and social services (HSS). These criteria should help describe and govern these networks as means for bringing on Schumpeterian innovations (i.e., product, process, organizational, ecosystem—adapting the market dimension—and input), balancing the triple-bottom line [73]. Our research conclusions are important because they equip elected politicians and senior public managers with empirical evidence about this networked strategy and its metagovernance [2,50,75]. We contribute here relevant criteria enabling the government of PSINs “socially penetrative” mode of governance [2], able to empower the state’s democratic legitimacy, efficiency, and effectiveness through collaboration [75].

Our work may be one of the first on the intersection between public services, innovation, networks, and sustainability. It features the internal, even hidden or latent, dimensions of the innovation networks (PSINs), aiming for sustainable goals through health and social public services innovations. These span from product and process innovations (e.g., pure clinical or technical innovations, concentrating on treatments for endangered local communities and ecosystem) to much more comprehensive efforts involving organizational and ecosystem innovations, originating from new combinations of technical and nontechnical input innovations, e.g., services for the cognitive and physically disabled elderly.

Knowing some of its benefits and pitfalls [2,10,13], we contribute to the metagovernance of HSS PSINs by modelling their dimensions, or attributes. These are critical for this “government of the governance” [2] (i.e., the metagovernance) of the collaborative strategy to innovate public services, especially if the strategy seeks a sustainable balance of goals. The latter include economic goals (efficiency and effectiveness), social goals (democratic legitimacy, trust, reputation), and environmental goals (reduction of resource depletion, avoidance of ecosystems’ breakdowns, circular economy) [76].

This metagovernance of HSS PSINs is crucial. It emphasizes the key question of how elected politicians and senior public managers can better govern (influence, command, and control [20]) public innovation networks. By definition, these are a “self-managed” form of governance [2]. PSINs are not isolated organizations, despite their obvious governance differences with traditional public or government structures. However, we found several remarks alluding to their hollowing out effect: the offload of the state’s or government’s functions on networks following a flawed understanding that they, on their own, would improve public services efficiency or performance.

HSS PSINs cannot act or survive alone. We agree with those authors who argue for the need of substantial political and public senior managerial involvement to secure PSIN democratic legitimacy and accountability [2]. Nevertheless, few of these calls described the specific “levers” politicians and managers can pull to secure the legitimacy of PSINs outcomes and innovations drawing on enhanced democracy and improved sustainability.

Therefore, we present here a precise and empirically validated set of political, institutional, operational, and administrative internal requirements for effective metagovernance of PSINs. We identified three major categories or dimensions of HSS PSINs—Social, Functioning Mode, and Actors, ordered by importance—that help effectively address the PSINs’ external requirements imposed by uncertainty, transaction costs, environmental or ecological sustainable development, and risk sharing [2].

Social is the most relevant of the three dimensions, featuring HSS PSINs as enhanced means to lower transaction costs and disseminate risks by engaging citizens and users. They collaborate in the design, production, and even delivery of new public services. We narrow the diversity of theoretical engagement types to, specifically, the collaboration of users and citizens in the assessment of user needs and their satisfaction pre- and post- delivery of new HSS services. How? By making users and citizens partners of the PSINs. What for? For the integration of products and services, the design of public services, and new ways of achieving HSS goals different from established and bureaucracy. All of them shape the specificities of HSS PSINs as agents for product, process, and input innovations.

To transform internal uncertainty derived from the different nature, knowledge, and involvement of HSS PSINs partners, they base their relationships on trust. Our theoretical framework predicted it, and our model’s Functioning mode empirically confirms it. Furthermore, the bindings are set on contracts, which clarifies the internal accountability of partners. Therefore, current HSS PSINs’ innovations, of either Schumpeterian types, are based on this combination of legal and behavioral bindings.

Although the third dimension, Actors, is noticeable, its weakness for HSS PSINs is surprising—for the Other PSINs, Actors is insignificant. Much is written about networks’ extended ability to involve collaborators other than civil servants, elected politicians, and public managers [18,75]. After all, PSINs are a form of organizing multi-actor collaborations for public innovation, spanning from public actors to for- and non-profit partners, including individual citizens [3,35].

Thus, we set our model’s indicators to grasp who the actual actors of HSS PSINs are, their motivation and commitment to collaborate, dedication, their relevance, and even their types of meetings. As expected, HSS PSINs actors include the citizens, committing to PSINs main duties (design, produce, and deliver new services). Other expected partners also become involved in HSS PSINs: universities and research centers.

Strikingly, we miss the decision-makers and the civil servants as actual actors of HSS PSINs. Their insignificant participation might well be predicted by Bell and colleagues [2], and others, alluding to the political and administrative challenges that PSINs pose for public officials and the hollowing out effect of the networked strategy. In addition, if HSS PSINs are to serve as instruments to enhance the ecological development of HSS, we miss other actors (e.g., for- and non-profits), too, who could contribute to make it happen.

HSS PSINs positively, though weakly, correlate with a combination of nontechnological, ad-hoc types of innovations [77,78,79,80]—mindset, idea, organizational, strategy, and policy innovations. They produce a mix of beneficial (e.g., higher participant satisfaction) and unexpected (e.g., higher design time) outcomes. The weak predictive strength of the PLS model for the HSS PSINs definitely points to the higher complexity of HSS PSINs. The different combinations of PSINs dimensions and their life cycle motivates the complexity. Furthermore, these innovations correlating with HSS PSINs are evidence of the Schumpeterian organizational and ecosystem innovations that networks bring on.

Our model allows the visualization of the different components of HSS PSINs, and how they linearly relate to their types of innovations and outcomes. The first learning from the metagovernance viewpoint is about the involvement of citizens. Through PSINs, they actively engage in designing, producing, and implementing new product–services systems, policies, strategies, and even mind-changing projects. This is a needed confirmation for elected politicians and senior management, with potential effects for policy- and decision-making.

However, the general model, based on the pooled sample of HSS respondents, could be misleading. It certainly presents the importance of each of the HSS PSINs components, which combined show the recipe for successful PSINs. However, the model’s predictive capability is weak under this pooled scenario. Does this mean the model is useless? Definitely, it is not.

Here is a second learning opportunity for policy- and decision-makers, and theorists. Rather than looking at HSS PSINs as a unique combination of components, from a pooled set of actors, the elected politicians and decision-makers must manage the different combinations of components, resulting from segmenting the actors. Our model is extremely efficient in presenting the effect of any of such combinations. For instance, the model can predict up to 67% of the innovations of HSS PSINs that, rather than growing in their early life cycle stages, constrain their dimensions to be more effective, which might be the case of networks with inexperienced partners. As another example, the model explains up to 55% of the innovations of HSS PSINs that wait until the relationships between the partners have matured, in a later life cycle stage of the PSIN—a likely consequence of too distant partners. As the last example, the model explains up to 70% of the innovations that HSS PSINs produce in their early stages and through an enlarged network, presumably of experienced partners.

The third learning opportunity is that the practical implications of the model introduce the possibility of playing with different scenarios for policy- and decision-making. Whether it is forward, from the components identified by the model to their resulting innovations, or backwards, from the estimated innovations back to their anteceding indicators, either way, elected politicians and decision-makers have now a tool to face the challenge posed by multi-agent networks as tools for improved efficiency, effectiveness, and democratic legitimacy. It is a dashboard-type tool which improves the impact analysis of the networked strategy.

The model and our research have certain limitations. Although our results are relevant in the context of Spanish public subsectors, including HSS, we encourage further research with larger samples and other geographies. Most importantly, the hidden scenarios and combinations, which can result in greater (or weaker) predictive strength of the model we have proposed, should be further investigated and fine-grained with larger samples of participants. We gathered our data during the first wave of the COVID-19 pandemic, when its effects might already have affected how partners organized and networked. Thus, it would be advisable to repeat this experiment under other conditions. Additionally, we were unable to detect significant differences between HSS PSINs and Other PSINs. We encourage further research to validate the generalizability of our model and its strengths. Additionally, we acknowledge the potential bias of the sample participants self-adscription. To prevent its unexpected effects, we controlled it during the pre- and post-administration of the survey, including, among others, controls for anonymity, oversized sample, and the PLS-SEM measurement tests.

To conclude, our research contributions are twofold. From a theoretical standpoint, it simplifies the complexity of components that earlier references listed as relevant for the development of public-network-based innovations. We correlated these components with the neo-Schumpeterian dimensions of innovation, including product, process, organizational, ecosystem, and input.

Second, PSINs are a relevant instrument for developing new ideas and facing complex projects with the aim of innovating services to address today’s societal problems. Very likely, they can be metagoverned and, with proper tools, enhance the state’s democratic legitimacy, efficiency, and effectiveness.

Being at this current early stage of the understanding of the networked strategy for public services innovation, we believe we have set here a sound ground to leverage future research by using our methodology and comparing our results with other geographies. Additionally, our PLS-path results can initiate other type of analyses to understand the dynamics of how PSINs produce their outcomes and innovations. We presume that agent-based models and other simulations can dynamically model our criteria. The interest might be in how individuals, partnering with PSINs, activate and repress their ties throughout idea generation, elaboration, and deployment. Similarly, the simulations can analyze the effect of the PSIN sizes and individual cognitive and emotional profiles in the successful creation of ideas and delivery of innovations. Finally, simulations might present credible and actionable visualizations of the social learning happening within the PSINs and its reflection in the wider context of public organizations.

## Figures and Tables

**Table 1:** Our sample population, by geographical scope and sector.

	HSS (^1^)	Other (^1^)	Count
Municipal	46	35	81 (38%)
Regional	33	39	72 (34%)
Cross-regional	9	9	18 (8%)
National	31	12	43 (20%)
Total	119	95	214

Notes: (**^1^**) Participants declaring their PSIN has a sustainable (economic, social, and/or environmental) orientation. HSS: Health and social services (aging, women, minorities, child- and youth-care services, and excluded populations). Other: Education, transportation and mobility, environment and urban, security, and employment.

**Table 2:** Path coefficients, significance, and VIF of the constructs for the health sector, and rest of the sectors, by their higher-order component.

				**HSS**		**Other**	
				**Path Coefficients**	**VIF**	**Path Coefficients**	**VIF**
2nd order variables							
Actors	Collaboration → Actors			0.892 ***	2.072	0.739 ***	1.856
	Motivation → Actors			0.173	1.246	0.156	1.025
	Relevance → Actors			0.052	1.608	0.569 **	1.556
	Types → Actors			0.073 ^	2.457	−0.275	2.137
Social	Engagement → Social			0.820 ***	1.172	0.762 ***	1.148
	Measurement → Social			0.064 *	1.033	0.147 ***	1.141
	Type-project → Social			0.350 ***	1.194	0.345 ***	1.116
3rd order variables							
PSINs	Actors → PSINs			0.120 ^	1.725	−0.201	1.668
	Functioning-mode → PSINs			0.293 **	1.198	0.382 ^	1.060
	Social → PSINs			0.804 ***	1.944	0.900 ***	1.701
Innovation	Life cycle → Innovation			−0.157 *	1.000	−0.147	1.000
	PSINs → Innovation			0.383 ***	1.000	0.273 **	1.000
Outcome	Life cycle → Outcome			0.150 *	1.000	0.059	1.000
	PSINs → Outcome			0.531 ***	1.000	0.381 ***	1.000
	**HSS**			**Other**			
	**R^2^**	**Q^2^**	**q^2^**	**R^2^**	**Q^2^**	**q^2^**	**Diff. of Paths**
**Innovation**	0.155	0.142	small	0.102	0.039	small	Not meaningful
**Outcome**	0.326	0.302	medium	0.146	0.055	small	Not meaningful

Notes: Significance: *** *p* < 0.001, ** *p* < 0.05, * *p* < 0.1. R^2^ values: 0.25, 0.50, and 0.75 indicate weak, moderate, and substantial predictive power [8,64]. Q^2^ values larger than 0 suggest that the model has predictive relevance of that construct [71,72,73]. q^2^ effect sizes: 0.02, 0.15, and 0.35 indicate small, medium, or large predictive relevance [73,74]. ^ nonsignificant indicators with loadings higher than 0.5 and theoretically valid.

## Appendix A

**Table A1:** Descriptive variables of our sample per geographical scope, gender, education, age, and type of organization.

	Non-Religious Private Agency, Foundation, Association or Entity	Not Working in Any or Working Autonomously	Public Administration, Agency or Entity	Religious Foundation, Association or Entity	Union	Count
**Municipal**	**24**	**4**	**40**	**2**		**70**
*Female*	*17*	*3*	*28*	*2*		*50*
Bachelor/engineering degree	6	2	11			19
26–45	4	1	4			9
46–65	2	1	7			10
Graduate/Master’s degree	11	1	16	2		30
26–45	10	1	7	2		20
46–65	1		9			10
Ph.D.			1			1
26–45			1			1
*Male*	*7*	*1*	*12*	*0*		*20*
Bachelor/engineering degree	3	1	5	0		9
26–45	1	1	3	0		5
46–65	2		2			4
Graduate/Master’s degree	4	0	5			9
26–45	3	0	0			3
46–65	1		5			6
Ph.D.			2			2
46–65			2			2
**Regional**	**24**	**1**	**32**	**1**	**0**	**58**
*Female*	*16*		*23*	*1*	*0*	*40*
Bachelor/engineering degree	6		13	1		20
26–45	3		6	1		10
46–65	3		7			10
Graduate/Master’s degree	9		8		0	17
26–45	5		5			10
46–65	4		3		0	7
High school/professional education	1		1			2
26–45	1		1			2
Ph.D.			1			1
46–65			1			1
*Male*	*8*	*1*	*9*			*18*
Bachelor/engineering degree	5	1	3			9
26–45	2	1	1			4
46–65	3		2			5
Graduate/Master’s degree	3		5			8
26–45	1		2			3
46–65	2		1			3
66–85			2			2
Ph.D.			1			1
46–65			1			1
**Cross-regional**	**8**	**1**	**4**			**13**
*Female*	*7*		*4*			*11*
Bachelor/engineering degree	3		1			4
26–45	1					1
46–65	2		1			3
Graduate/Master’s degree	4		3			7
26–45	3		1			4
46–65	1		2			3
High school/professional education			0			0
46–65			0			0
*Male*	*1*	*1*	*0*			*2*
Bachelor/engineering degree	0		0			0
26–45			0			0
46–65	0					0
Graduate/Master’s degree	1		0			1
26–45	1		0			1
High school/professional education		1				1
46–65		1				1
**National**	**27**	**3**	**8**		**2**	**40**
*Female*	*19*	*3*	*5*		*2*	*29*
Bachelor/engineering degree	8		1		2	11
26–45	4		1		2	7
46–65	4		0			4
Graduate/Master’s degree	11	2	4			17
26–45	9	2	1			12
46–65	2		3			5
High school/professional education		1				1
46–65		1				1
*male*	*8*		*3*			*11*
Bachelor/engineering degree	3		1			4
26–45	3					3
46–65			1			1
Graduate/Master’s degree	4		2			6
26–45	2					2
46–65	2		2			4
High school/professional education	1					1
46–65	1					1
Total	83	9	84	3	2	181

Bold number summarizes the figures up to the next bold title, same with italics.

**Table A2:** Research items per construct and measurement scales.

Construct	Item	Item Question	Scale
**Loadings (Mode A)**			
INNOVATION	INNFOR01[IF01]	The main type of goal of this group you are describing was… a technical, market or industrial innovation—e.g., prototype, tender, patent, regulation, or norm	Y/N
	INNFOR01[IF02]	The main type of goal of this group you are describing was… a non-technical, service innovation OR a combination of technical and non-technical innovation—e.g., a policy, improve or creation of a service, digitalization, new organization, new process	Y/N
	INNFOR03[IF03]	The innovation/s of the group you are describing were mainly… planned (step-by-step) innovation, with little deviances from the plan	Y/N
	INNFOR03[IF04]	The innovation/s of the group you are describing were mainly… unplanned (spontaneous) innovation	Y/N
	INNFOR03[IF05]	The innovation/s of the group you are describing were mainly… a combination of planned and unplanned	Y/N
	INNTYP01[IT01]	Please, describe the scale of the changes produced by that group in… a product	Incremental (update)/Radical change or creation/Not applicable
	INNTYP01[IT02]	Please, describe the scale of the changes produced by that group in… a process	Incremental (update)/Radical change or creation/Not applicable
	INNTYP01[IT03]	Please, describe the scale of the changes produced by that group in… an organization or group of people	Incremental (update)/Radical change or creation/Not applicable
	INNTYP01[IT04]	Please, describe the scale of the changes produced by that group in… a concept or idea	Incremental (update)/Radical change or creation/Not applicable
	INNTYP01[IT05]	Please, describe the scale of the changes produced by that group in… a strategy or policy	Incremental (update)/Radical change or creation/Not applicable
	INNTYP01[IT06]	Please, describe the scale of the changes produced by that group in… how people usually think	Incremental (update)/Radical change or creation/Not applicable
	INNTYP01[IT07]	Please, describe the scale of the changes produced by that group in… how things are traditionally done	Incremental (update)/Radical change or creation/Not applicable
	INNTYP01[IT09]	Please, describe the scale of the changes produced by that group in… a service	Incremental (update)/Radical change or creation/Not applicable
	ISSO01[IS01]	The type of problems that group wanted to solve were… mostly well identified and allowing a rather well-defined solution	Y/N
	ISSO01[IS02]	The type of problems that group wanted to solve were… mostly un-identified, and needing experimentation and an unclear combination of solutions or approaches	Y/N
	ISSO02[SO01]	The innovation your group aimed for was… adopted or seen somewhere else	Y/N
	ISSO02[SO02]	The innovation your group aimed for was… produced or originated in the group	Y/N
FEELING	ACTORS06[AC26]	In that group you are describing, you felt… less committed to the other agents in the group than if you had worked with them outside it	1–5
	ACTORS06[AC27]	In that group you are describing, you felt… your group did not really consider the users’ preferences	1–5
	ACTORS06[AC28]	In that group you are describing, you felt… your group was more focused on performance than innovation	1–5
INTENSITY	ACTORS07[AC29]	The group you have been describing in this survey is… a permanent (i.e., intended to last indefinitely) group	List(radio)
	ACTORS07[AC30]	The group you have been describing in this survey is… a temporary (i.e., time-limited) group	List(radio)
LIFE-CYCLE	STAGE01[ST01]	What is the stage of that group to develop public services?… In the early stages, still organizing who, what, when, etc.	List(radio)
	STAGE01[ST01]	What is the stage of that group to develop public services?… Mid-stage, we are progressing now but still have some roughness in our progress	List(radio)
	STAGE01[ST02]	What is the stage of that group to develop public services?… Mature stage, we have achieved some main successes and we are flowing	List(radio)
	STAGE01[ST02]	What is the stage of that group to develop public services?… End stage, the network is already stopping because it achieved its goals	List(radio)
	STAGE01[ST02]	What is the stage of that group to develop public services?… Decline stage, only a few or no one really cares about the network	List(radio)
MEASUREMENT	MODORG04[MO23]	In the innovations or developments produced by that group you are describing, did you measure…? Outputs like productivity, efficiency, units produced or similar	Y/N
	MODORG04[MO24]	In the innovations or developments produced by that group you are describing, did you measure…? Outcomes like costs, returns, value added, revenue	Y/N
	MODORG04[MO25]	In the innovations or developments produced by that group you are describing, did you measure…? Indicators of relations like equality, justice, inclusion, service quality	Y/N
RELATIONSHIP	ACTORS04[AC18]	Which was the most common type of relationships among agents in your group? Bilateral meetings	List (radio)
	ACTORS04[AC19]	Which was the most common type of relationships among agents in your group? Multi-party meetings	List (radio)
RELEVANCE	ACTORS03[AC12]	Beyond the intensity, how would you rate the importance of the contribution of universities to achieve that group’s goals?	1–5
	ACTORS03[AC13]	Beyond the intensity, how would you rate the importance of the contribution of Public administrations to achieve that group’s goals?	1–5
	ACTORS03[AC14]	Beyond the intensity, how would you rate the importance of the contribution of Services firms to achieve that group’s goals?	1–5
	ACTORS03[AC15]	Beyond the intensity, how would you rate the importance of the contribution of Industrial or agricultural companies to achieve that group’s goals?	1–5
	ACTORS03[AC16]	Beyond the intensity, how would you rate the importance of the contribution of NGOs, foundations, associations and unions to achieve that group’s goals?	1–5
	ACTORS03[AC17]	Beyond the intensity, how would you rate the importance of the contribution of Users/citizens to achieve that group’s goals?	1–5
**Weights (Mode B)**			
COLLABORATION	ACTORS05[AC20]	That group you are describing… included end users/citizens in idea generation or prototyping sessions	1–5
	ACTORS05[AC21]	That group you are describing… included end users/citizens in services or processes co-design/co-implementation	1–5
	ACTORS05[AC22]	That group you are describing… included end users in the analysis of data on their experiences	1–5
	ACTORS05[AC23]	That group you are describing… included other agents (consultants, technical staff or any other) in idea generation or prototyping sessions	1–5
	ACTORS05[AC24]	That group you are describing… included other agents (consultants, technical staff or any other) in services or processes co-production/co-implementation	1–5
	ACTORS05[AC25]	That group you are describing… worked with users’ representatives (e.g., NGOs, associations) more than with individual end users or citizens	1–5
ENGAGEMENT	MODORG05[MO27]	Did your group…? evaluate the actual engagement of users/citizens	1–5
	MODORG05[MO28]	Did your group…? assess user/citizen satisfaction with the service or process, pre- and post- innovation	1–5
	MODORG05[MO29]	Did your group…? improve the assessment of the needs of users/citizens because they were de-facto members of the network	1–5
	MODORG05[MO30]	Did your group…? study the needs of users/citizens using market research techniques	1–5
FUNCTIONING-MODE	MODORG06[MO31]	Did your group arrange…? Around a central entity	Y/N
	MODORG06[MO32]	Did your group arrange…? Based on trust, reputation, and/or earlier collaboration among some main entities	Y/N
	FUNCTI01[FU01]	That group you are describing was… part of a formal plan (e.g., tender, norm)	Y/N
	FUNCTI01[FU02]	That group you are describing was… emerged spontaneously, not related to any formal plan	Y/N
	FUNCTI02[FU03]	That group functioned… With a vertical, hierarchical, or top-down mode	Y/N
	FUNCTI02[FU03]	That group functioned… with a horizontal, collaborative, or bottom-up mode	Y/N
	FUNCTI03[FU06]	In that group you are describing, there was… trust instead of bureaucracy	1–5
	FUNCTI03[FU07]	In that group you are describing, there was… collaboration instead of orders	1–5
	FUNCTI03[FU08]	In that group you are describing, there were… all agents managed together the risk of disclosure	1–5
	FUNCTI03[FU09]	In that group you are describing, there were… contracts formalized the arrangements between agents	1–5
	FUNCTI04[FU10]	The role of the main public agent in that group was… proponent or central authority of the project	Y/N
	FUNCTI04[FU11]	The role of the main public agent in that group was… second to a proposing non-public agent, but actively supporting and facilitating the project	Y/N
	FUNCTI04[FU12]	The role of the main public agent in that group was… passively supporting private agents	Y/N
	FUNCTI04[FU13]	The role of the main public agent in that group was… no public agents	Y/N
MOTIVATION	MOTIVA01[MO01]	You decided to embark in your last group to develop services due to… your manager suggested it	Y/N
	MOTIVA01[MO02]	You decided to embark in your last group to develop services due to… the group aimed to develop or innovate a particular service	Y/N
	MOTIVA01[MO03]	You decided to embark in your last group to develop services due to… your unit is dedicated to this type of projects	Y/N
	MOTIVA01[MO04]	You decided to embark in your last group to develop services due to… you were following confirmed political guidelines	Y/N
	MOTIVA01[MO05]	You decided to embark in your last group to develop services due to… it was an open group willing to admit everyone interested	Y/N
OUTCOME	OUTCOM01[OU01]	Thinking on that last group of public service innovation, how would you rate its outcomes?… design time	1–5
	OUTCOM01[OU02]	Thinking on that last group of public service innovation, how would you rate its outcomes?… ability to target user needs	1–5
	OUTCOM01[OU03]	Thinking on that last group of public service innovation, how would you rate its outcomes?… number of citizens able to access the service	1–5
	OUTCOM01[OU04]	Thinking on that last group of public service innovation, how would you rate its outcomes?… user experience of the service	1–5
	OUTCOM01[OU05]	Thinking on that last group of public service innovation, how would you rate its outcomes?… implementation time	1–5
	OUTCOM01[OU06]	Thinking on that last group of public service innovation, how would you rate its outcomes?… user access to information	1–5
	OUTCOM01[OU07]	Thinking on that last group of public service innovation, how would you rate its outcomes?… employee satisfaction/working conditions	1–5
	OUTCOM01[OU08]	Thinking on that last group of public service innovation, how would you rate its outcomes?… service quality	1–5
	OUTCOM01[OU09]	Thinking on that last group of public service innovation, how would you rate its outcomes?… Procedures	1–5
	OUTCOM01[OU10]	Thinking on that last group of public service innovation, how would you rate its outcomes?… Costs	1–5
	OUTCOM01[OU11]	Thinking on that last group of public service innovation, how would you rate its outcomes?… fit of services and technical requirements (time, resources, effectiveness, etc.)	1–5
TYPE-PROJECT	MODORG01[MO01]	Deepening in the goals of that group you are describing, you and the rest of its members aimed for… the design of a public service	1–5
	MODORG01[MO02]	Deepening in the goals of that group you are describing, you and the rest of its members aimed for… the delivery of a public service	1–5
	MODORG01[MO03]	Deepening in the goals of that group you are describing, you and the rest of its members aimed for… a private product or service	1–5
	MODORG01[MO04]	Deepening in the goals of that group you are describing, you and the rest of its members aimed for… the rationalization of a process (e.g., of production)	1–5
	MODORG01[MO05]	Deepening in the goals of that group you are describing, you and the rest of its members aimed for… the adoption of a technical system or a process	1–5
	MODORG01[MO06]	Deepening in the goals of that group you are describing, you and the rest of its members aimed for… new paths to achieve the group’s goals, free from the established or bureaucratic procedures	1–5
	MODORG01[MO07]	Deepening in the goals of that group you are describing, you and the rest of its members aimed for… the integration of products in services	1–5
TYPES	ACTORS01[AC01]	What was the intensity of the participation of universities in that network you are describing?	1–5
	ACTORS01[AC02]	What was the intensity of the participation of Research laboratories or institutes in that network you are describing?	1–5
	ACTORS01[AC03]	What was the intensity of the participation of Local public administration in that network you are describing?	1–5
	ACTORS01[AC04]	What was the intensity of the participation of Regional public administration in that network you are describing?	1–5
	ACTORS01[AC05]	What was the intensity of the participation of National public administration in that network you are describing?	1–5
	ACTORS01[AC06]	What was the intensity of the participation of Consultant firms in that network you are describing?	1–5
	ACTORS01[AC07]	What was the intensity of the participation of Financial services firms in that network you are describing?	1–5
	ACTORS01[AC08]	What was the intensity of the participation of Services firms (any other type) in that network you are describing?	1–5
	ACTORS01[AC09]	What was the intensity of the participation of Industrial, construction, agricultural industries in that network you are describing?	1–5
	ACTORS01[AC10]	What was the intensity of the participation of NGOs, foundations, associations and unions in that network you are describing?	1–5
	ACTORS01[AC11]	What was the intensity of the participation of Users/citizens in that network you are describing?	1–5
WICKED	MODORG02[MO09]	Speaking of social problems or needs, which of the following were addressed by that group you are describing?… Health	Y/N
	MODORG02[MO10]	Speaking of social problems or needs, which of the following were addressed by that group you are describing?… Aging	Y/N
	MODORG02[MO11]	Speaking of social problems or needs, which of the following were addressed by that group you are describing?… Education/training	Y/N
	MODORG02[MO12]	Speaking of social problems or needs, which of the following were addressed by that group you are describing?… Transportation and mobility	Y/N
	MODORG02[MO13]	Speaking of social problems or needs, which of the following were addressed by that group you are describing?… Environment and urban problems	Y/N
	MODORG02[MO14]	Speaking of social problems or needs, which of the following were addressed by that group you are describing?… Security	Y/N
	MODORG02[MO15]	Speaking of social problems or needs, which of the following were addressed by that group you are describing?… Employment	Y/N
	MODORG02[MO16]	Speaking of social problems or needs, which of the following were addressed by that group you are describing?… Women/minorities/excluded populations	Y/N
	MODORG02[MO17]	Speaking of social problems or needs, which of the following were addressed by that group you are describing?… Childhood/youth	Y/N

Note: dropped items during validity and reliability tests of first-order components are crossed.
